# Chemiluminescence systems; do all lead to same results in prolactin analysis?

**DOI:** 10.1186/s40200-017-0305-7

**Published:** 2017-06-05

**Authors:** Marjan Rahnamaye Farzami, Mehri Aliasgharpour

**Affiliations:** Department of Biochemistry, Reference Health Laboratory, Ministry of Health & Medical Education, Tehran, Iran

**Keywords:** Prolactin, Chemiluminescence, Pituitary gland, Gel filtration, Chromatography, Prolactinoma

## Abstract

**Background:**

Human prolactin (PRL) is a hormone that is mainly secreted by lactotroph cells of the anterior pituitary gland and is involved in many biological processes including lactation and reproduction. Prolactin level may be determined quantitatively in serum by many different systems including chemiluminescence systems. However, comparison of the measured values between systems is difficult.

**Methods:**

The prolactin values obtained and compared in two chemiluminescence systems; AUTOBIO DIAGNOSTICS MICROPLATE LUMOMETER and LIAISON XL Analyzer using BioRad tri level serum quality control materials and serum samples from *n* = 44 female patients; (Age mean & range) = (33: 21–65) years.

**Results:**

Obtained PRL mean and range in Autobio and Liason systems were X = 414.8 ± 230.0; Range: 25.7–980.9 μlU/mL & X = 391.7 ± 225.6; Range: 26.0–991.4 μlU/mL respectively. Both system’s results were correlated (Pearson product moment correlation *r* = 0.97 at *p* = 0.01 and Regression Analysis).

**Conclusion:**

Because of the differences between CLIA systems the authors conclude laboratories measuring range for PRL be accomplished on particular analyzer and verified against reference intervals stated by the manufacture. More importantly, consecutive PRL level determinations and patients follow up should be performed on only one analyzer rather than different analyzers. In this regard, mentioning the method and system type on the final laboratory reports become important and verify that a laboratory considers the clinical aspects of laboratory request as well as the quality assurance in performing the PRL determination.

## Background

In the human genome prolactin (PRL) molecule is arranged in a single chain of amino acids with three intra-molecular disulfide bonds between six cysteine residues and is synthesized and secreted by the lactotroph cells of the anterior pituitary gland [1]. Three major variant of PRL can be found in the blood: monomeric, big and macroprolactin . Multiple circulating variants of PRL are identifiable on Gel Filtration Chromatography [2]. The monomeric form has a molecular mass of ~23 kDa [3] and accounts for most of the PRL present in normal individuals. Prolactin is normally present in low amounts in both men and non-pregnant women and mainly its level is regulated by dopamine [4].

Regardless of hypersecretion of prolactin with idiopatic reasons that may lead to hyperprolactinemia due to macroprolactinemia, the most common cause of elevated PRL level (hyperprolactinemia) is prolactin-producing adenomas or prolactinomas [5–7]. Adenomas occur in both men and women with abnormal reproductive function or with galactorrihea. However, it is more commonly diagnosed in women who are less than 50 years old. Furthermore, most adenomas occur sporadically and rarely in families as a condition called the multiple endocrine neoplasia type 1 syndrome [8].

The PRL level is measured in a blood serum sample and increased result can range from elevated to multiple times the upper limit of normal. Larger adenomas cause higher PRL levels and elevated concentrations are found in up to 17% of such cases [5, 9, 10]. There are other causes of increased PRL level in serum such as use of various drugs, compression of the pituitary stalk, presence of macroprolactin, renal failure, cirrhosis or idiopathic hyperprolactinemia are diagnoses that may increase PRL level [9, 10].

Different methods are used to measure PRL level. They are mostly immunoassay based such as RIA, ELISA, CLIA, ECL. Studies have indicated that Gel Filtration Chromatography is the gold standard for quantification of PRL molecular forms in serum. However, it is slow, costly and labour intensive [11, 12].

Although precise laboratory techniques demonstrating hyperprolactinemia are essential for the accurate diagnosis,treatment and monitoring of patients during medical therapy or post surgery, the comparison of the measured values among different systems are difficult. Because the methods are not identical in different laboratories and the reference ranges are different for each. This is one of the main challenges in PRL measurements among laboratories.

In the present study we compared the results of two chemiluminescence immunoassay (CLIA) methods to determine serum prolactin level in 44 female patients serum sample(age range 21–65 years).

## Methods

Serum samples for PRL determination from female patients (*n* = 44; age mean = 33, range = 21–65 years) were collected using standard collecting procedures during fall 2015 at the Reference Health laboratory of Iran, splitted and kept frozen for maintaining long term stability until the analysis. Prolactin level in selected specimens were distributed through claimed analytical range of both assays and were first verified before beginning the comparison tests. In both methods calibrators were traceable to WHO 3rd International Reference preparation (85/500).

Determinations were performed using two CLIA systems and kits; AUTOBIO DIAGNOSTICS MICROPLATE LUMOMETER (CLIA-China;REF-CL1103–2) and LIAISON XL Analyzer (CLIA-Italy;REF-312171). BioRad quality control materials (3 levels) were also ran. Obtained values from two assay methods were compared using Descriptive, Pearson product moment correlation and Regression analysis for the patients samples.

## Results

Obtained PRL values for patients sample (*n* = 44) on two systems (Autobio & Liaison) were as in Table 1. Furthermore, Bio-Rad tri level control materials results were in the stated ranges of the brochure considering the mentioned target points.

**Table 1:** Descriptive analysis of PRL in patients samples on CLIA Kits

Patient (*n* = 44)	Autobio	Liaison
PRL; Mean (μlU/mL)	414.8 ± 230.0	391.7 ± 225.6
PRL; Range	25.7–980.9	26.0–991.4

**Fig. 1:**
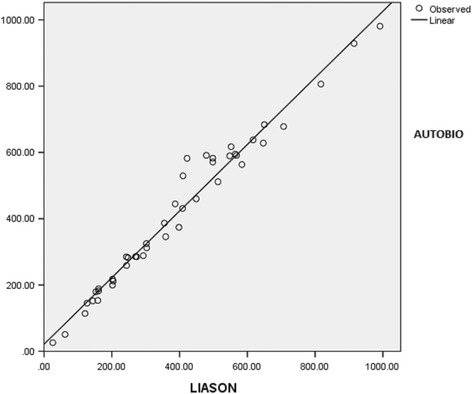
Regression analysis of PRL obtained values for patients serum samples in two CLIA systems. * Dependent variable: AUTOBIO (Y = 21.2 + 1.01X)

Pearson product moment correlation and Regression analysis for PRL concentrations in patients sample (*n* = 44) indicated a significant correlation at *p* = 0.01 (*r* = 0.97) for two CLIA systems (Fig. 1).

## Discussion

**Fig. 2:**
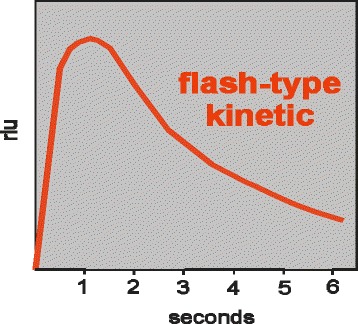
Flash-type kinetic (Liason)

**Fig. 3:**
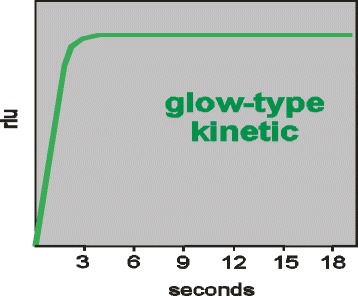
Glow- type kinetic (Autobio)

Recently the main measurement systems of many hormones including PRL has shifted from RIA methods to non RIA methods especially to Luminescence ones. However, among the commercially available PRL assays, results vary widely. Chemiluminescence assays are preferred methods used in clinical laboratories because they have better sensitivity, and produce low background. In addition, the source of energy to excite the molecule is a chemical reaction frequently oxidation. To enhance the luminescent signal, some enzymes and their substrates react within a short period of time and emit a single, brief flash of luminescence. Therefore, the microplate must be measured immediately after dispensing of substrate solution. Other enzymes and substrates react to emit a stable glow over several minutes or hours. The differences between the two form of chemiluminescence are shown in Figs. 2, 3.

The systems in our present study for PRL concentration determinations (*n* = 44) were chemiluminescence AUTOBIO DIAGNOSTICS MICROPLATE LUMOMETER and LIAISON XL . The two assay methods were different in that Autobio Lumometer system produces glow type luminescence while Liaison XL Analyzer produces flash type of luminescence. Analysis of the data using Pearson product moment correlation and Regression for PRL concentrations determination indicated that results at *p* = 0.01 for the two CLIA systems were correlated(*r* = 0.97). Higher mean values of PRL in Autobio system and observed systematic difference on the graph (Fig. 1), could be explained by different avidity and affinity properties of antibodies selected by the manufactures. Furthermore, by different reaction times and kinetics used in designing two assay procedures which itself explains different reference ranges for each kit systems. Different studies have also been indicated that different factors are effective in obtained PRL values among different systems [13–15]. In addition, studies have been shown that presence of macroprolactin may lead to false higher PRL values leading to errors in clinical decision makings [16–18] and it is suggested that patients with hyperprolactinemia should be tested for macroprolactin as well [16]. Establishment of external quality assessment programs has also been shown [19] to be a useful tool to monitor the performance of prolactin measuring assays in enrolled laboratories.

## Conclusion

Based on our obtained results and observed differences between two CLIA systems the authors conclude that laboratories measuring range for PRL should be accomplished on the particular analyzer and verified against stated reference interval by the manufacture. More importantly, consecutive patients PRL level determinations and follow ups should be performed on same analyzer. In this regard, mentioning the method and system type used for PRL determination on the final laboratory report becomes important and verifies that a laboratory considers the clinical aspects of laboratory requests as well as the quality assurance in performing the PRL determination.

## Abbreviations

CLIA: Chemiluminescence immunoassays
ELISA: Enzyme linked immunosorbent assay
EQAS: External quality assessment scheme
MRI: Magnetic resonance imaging
PRL: Prolactin
RIA: Radio Immunoassays
